# Inter-Rater Reliability of Subarachnoid Hemorrhage Radiological Grading Scales: A Systematic Review and Meta-Analysis

**DOI:** 10.3390/jcm15082899

**Published:** 2026-04-10

**Authors:** Daria Dmitrievna Dolotova, Tatyana Alexandrovna Solominova, Natalia Alexeevna Polunina, Evgenia Romanovna Blagosklonova, Natalya Sergeevna Plyusova, Ganipa Ramazanovich Ramazanov, Rustam Shakhismailovich Muslimov, Maxim Vladimirovich Solominov, Andrey Vasilevich Gavrilov

**Affiliations:** 1 Research and Clinical Institute for Pediatrics Named After Yuri Veltischev, Pirogov Russian National Research Medical University, 125412 Moscow, Russia; 2 Research Department, Gammamed-Soft, Ltd., 127473 Moscow, Russia; tatiana.solominova@gammamed.ru (T.A.S.); evgenia.blagosklonova@gammamed.ru (E.R.B.); maksim.solominov@gammamed.ru (M.V.S.); andrey.gavrilov@gammamed.ru (A.V.G.); 3 Department of Fundamental Neurosurgery, Pirogov Russian National Research Medical University, 117513 Moscow, Russia; polunina_na@rsmu.ru; 4 Sklifosovsky Research Institute for Emergency Medicine, Moscow Health Department, 129090 Moscow, Russia; plyusovans@sklif.mos.ru (N.S.P.); ramazanovgr@sklif.mos.ru (G.R.R.); muslimovrsh@sklif.mos.ru (R.S.M.); 5 Scobeltsyn Nuclear Physics Research Institute, Lomonosov Moscow State University, 119991 Moscow, Russia

**Keywords:** subarachnoid hemorrhage, computed tomography, inter-rater reliability, Fisher scale, Hijdra scale, meta-analysis

## Abstract

**Background**: Subarachnoid hemorrhage (SAH) has high mortality and disability rates. The timely and precise assessment of SAH severity is of critical importance in predicting life-threatening complications. Several CT-based radiological grading systems have been proposed, but a comprehensive meta-analysis of their inter-rater reliability (IRR) has not been conducted. **Methods**: This study followed the guidelines of Preferred Reporting Items for Systematic Reviews and Meta-Analyses (PRISMA). Two authors performed a systematic search of original articles in the PubMed database. Methodological quality of the studies was assessed using the Quality Appraisal of Reliability Studies (QAREL). Meta-analyses of Cohen’s kappa and intra-class correlation coefficient (ICC) were performed using R packages “metafor” and “meta”. **Results**: A systematic literature analysis was performed for twenty articles that met the inclusion criteria. The methodological quality was moderate in 14 of 20 studies; five studies were of low quality. Only eight articles were suitable for meta-analysis. Cohen’s kappa of the binarized Fisher scale was 0.85 (95% CI 0.70–0.93), though it was based on only two studies and 109 patients. The Hijdra scale had an ICC of 0.75 (95% CI 0.29–0.93). The original and modified Graeb scales proposed for the assessment of concomitant intra-ventricular hemorrhage demonstrated ICC of 0.83 (95% CI 0.59–0.94) and 0.93 (95% CI 0.84–0.97), respectively. For other scales, meta-analysis was not possible due to incomplete reporting or single evaluations. **Conclusions**: The current evidence on IRR of radiological grading scales for SAH is limited, emphasizing the need for further high-quality research to validate their reliability and clinical applicability.

## 1. Introduction

The burden of acute cerebrovascular accidents remains high in developed countries [1,2,3]. Mortality and disability risks are several times higher in hemorrhages [4]. Subarachnoid hemorrhage (SAH), being of either non-traumatic (85% of cases) or traumatic etiology [5,6,7,8,9,10], is characterized by a 30-day mortality rate of 42% and a 50% probability of developing disability in surviving patients [11,12,13]. The reason for this is the peculiarity of its course [14]. The presence of blood in subarachnoid spaces triggers a cascade of reactions that lead to cerebral vasospasm, which is a narrowing of the arterial walls [15]. In more than 60% of patients, the development of cerebral vasospasm causes delayed cerebral ischemia manifested via transient or permanent neurological deficit [16,17].

Due to the heightened risk of complications, a rapid and accurate assessment of SAH severity is a priority. The primary tool for this purpose is brain computed tomography (CT) [18]. It provides a swift diagnosis of the hemorrhage type, as well as an assessment of its severity [19]. Manual assessment scales applied to CT images have been proposed to standardize the evaluation of SAH and concomitant intra-ventricular hemorrhage (IVH) [20,21,22,23,24,25,26]. Most scales demonstrate a statistically significant relationship between the score and the treatment outcome, along with the risks of delayed ischemia, hydrocephalus, and other complications [27,28,29,30,31]. Furthermore, they can also be used to choose the most appropriate patient management tactics [32,33].

Despite a plethora of scientific publications on radiological scales, most clinical guidelines contain none [34,35,36,37,38]. Those that suggest qualitative assessment of CT images [33,39,40,41,42,43] recommend the Fisher scale [24] in combination with clinical condition scales such as the Glasgow coma scale and Hunt–Hess scale [41]. Other scales, including Hijdra [22], original and modified Graeb [20,21], modified Fisher [23], and others [44,45,46,47,48,49], are mentioned less frequently and primarily in research contexts.

An important attribute of any scale in medicine is its inter-rater reliability (IRR), which reflects the level of agreement among experts when assigning a particular scale gradation to a clinical case [50]. In clinical practice, scales with low IRR may lead to erroneous tactical and treatment decisions, thereby putting the patient’s life at risk. For most of the aforementioned scales, IRR has been evaluated [45,48,51]. Far fewer studies have aimed at comparative analyses of IRR across various scales [20,46,52], concluding that one scale may be preferable to others. To date, there has been no systematic literature review on the topic.

Based on the above, the aim of this study is to systematically identify, evaluate, and synthesize existing evidence regarding the IRR of radiological grading scales used in the assessment of SAH and IVH on computed tomography in order to determine their suitability for clinical application.

## 2. Materials and Methods

### 2.1. Search and Selection of Publications

A systematic search was conducted for scientific publications on the IRR of manual scales for SAH severity assessment based on radiological images, using PubMed (NCBI), a comprehensive and authoritative resource, containing over 40 million citations and abstracts focused on biomedical and clinical topics. The selection was primarily guided by the following factors: (1) extensive and specialized indexing in medical subjects through medical subject headings (MeSH); (2) full Boolean logic support, enabling more precise and sensitive literature retrieval; (3) free access, ensuring reproducibility of search results.

The search was performed between June and November 2025. A selection was made of articles published in English, with publication dates up to 31 August 2025. Given the absence of systematic reviews and meta-analyses on the subject, only original scientific articles were consulted.

The population under study comprised adults (over 18 years old) with diagnosed SAH and/or IVH confirmed by CT data. The inclusion and exclusion criteria followed the studies, data, methods, and outcomes (SDMO) framework that is recommended for methodological systematic reviews [53,54] and were formulated as follows:

Inclusion criteria: (a) studies involving human subjects diagnosed with SAH and/or IVH, assessed via cranial CT; (b) studies explicitly reporting IRR metrics (e.g., kappa, intra-class correlation coefficients (ICC)) for one or more radiological grading scales.

Exclusion criteria comprised: (a) studies that do not report inter-rater reliability data (or only report descriptive or qualitative assessments without quantitative IRR); (b) reviews, editorials, commentaries, or case reports; (c) studies using imaging modalities other than CT (e.g., MRI); (d) studies involving a pediatric population.

The following query was employed: (((“subarachn*” OR “intraventricul*” OR “intracerebr*”) AND (“hemorrhage” OR “haemorrhage”OR”extension”)) OR “aneurysmal” OR “aSAH” OR “SAH” OR “IVH” OR “ICH” OR “DCI” OR ((“subarachoid” OR “extravasated” OR “ventricular” OR “intraventricular” OR “cisternal” OR “intracerebral” OR “subarachnoid”) AND (“blood” OR (“hemorrhage” OR “haemorrhage”)))) AND (((“radiological” OR “CT” OR “computed tomogram” OR “computed tomography”) OR (“Fisher” OR “original Fisher” OR “mFisher” OR “modified Fisher” OR “Graeb” OR “original Graeb” OR “Modified Graeb” OR “mGraeb” OR “mGS” OR “Hijdra” OR “modified Hijdra” OR “Claassen” OR “BNI” OR “LeRoux” OR “Slice”) OR (“grading” OR “quantitative” OR “new” OR “comprehensive”)) AND (“scale” OR “score” OR “featur*” OR “grad*” OR “method” OR “scoring system” OR “sum score”)) AND ((“inter-observer” OR “interobserver” OR “inter-rater” OR “interrater” OR “inter-expert” OR “inter-”) AND (“agreement” OR “reliability” OR “variability”) OR (“ICC” and “kappa”)).

**Figure 1:**
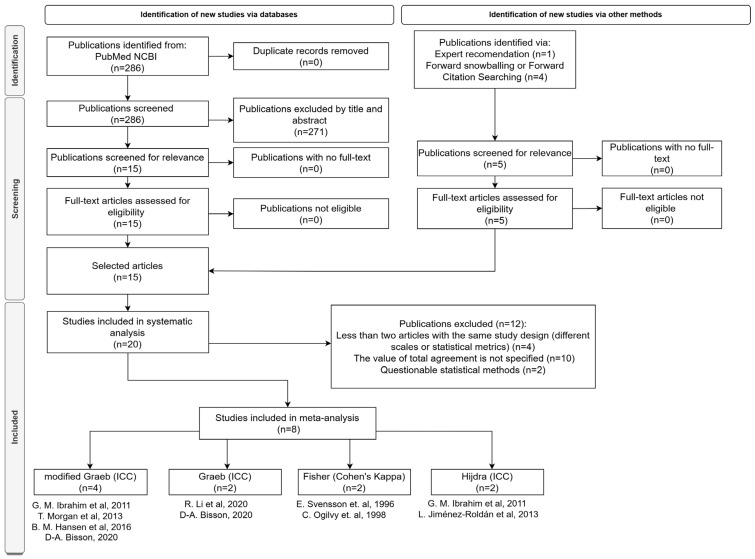
Flow diagram meeting PRISMA 2020 criteria (T. Morgan, et al., 2013 [20]; R. Li et al., 2020 [48]; L. Jiménez-Roldán, 2013 [56]; E. Svensson et al., 1996 [57]; C.S. Ogilvy et al., 1998 [58]; G.M. Ibrahim et al., 2011 [59]; B.M. Hansen et al., 2016 [60]; D.-A. Bisson et al., 2020 [61]).

The reporting of this systematic review was guided by the standards of the Preferred Reporting Items for Systematic Review and Meta-Analysis (PRISMA) Statement [55]. The systematic review was not registered in PROSPERO or any other database; the full review protocol is available in Supplementary File S1. Additionally, the PRISMA 2020 Checklist (Table S1) and the Abstracts Checklist (Table S2) are provided in the Supplementary Materials. We have obtained 286 articles, and two authors (T.A.C. and N.A.P.) screened the database search results independently, assessing articles based on the title and abstract according to the inclusion and exclusion criteria. At this stage, 271 of 286 articles did not meet the eligibility criteria (Figure 1).

One paper was added on the recommendation of an expert and four works were included after analyzing the reference lists from the already selected publications. A total of 20 sources were included in the systematic review, with the meta-analysis conducted on the results of 8 articles.

### 2.2. A Brief Description of Radiological Scales

One of the oldest and most common assessment methods is the Fisher scale, proposed in 1980 [24]. Its convenience and simplicity of assessment, along with the accuracy of diagnostic criteria and high prognostic significance, make the scale so popular [62]. For instance, grade 3 on the Fisher scale is statistically associated with a high probability of developing cerebral vasospasm [24,63]. However, despite its simplicity, the scale has a rather low IRR, confirmed by some researchers [46,47,64]. In this regard, J. Frontera et al. proposed a modified Fisher scale (mFisher) in 2006 [23]. This modified version also retains four grades but places a greater emphasis on intra-ventricular hemorrhages (IVHs) (Table 1).

The researchers claim that the Fisher scale they modified has a greater predictive ability for assessing the risks of vasospasm, delayed ischemia, and adverse outcome compared to the original one [64,65].

Other scales with a small number of gradations are those by Claassen [45] and the Barrow Neurological Institute (BNI) [46,47]. The Claassen scale is very similar to the modified Fisher scale but considers the presence of blood only in the brain’s lateral ventricles. In contrast, the BNI scale does not assess the presence of blood in the ventricles and proposes to use maximum thickness to assess SAH severity.

Some scales aim at a detailed assessment of the severity of concomitant IVH. The most common of them is the Graeb scale proposed by a group of authors in 1982 [21]. In 2013, its modification (mGraeb scale) was also offered, embracing IVH localization and volume as well as the distribution of IVH in the horns of the lateral ventricles and their enlargements (Table 2).

Less widely employed are the LeRoux [49], IVH score [66], and Slice [48] scales. It should be also noted that, when compared to others, these scales have fewer publications describing their inter-rater reliability [46,48,56,64,65].

**Figure 2:**
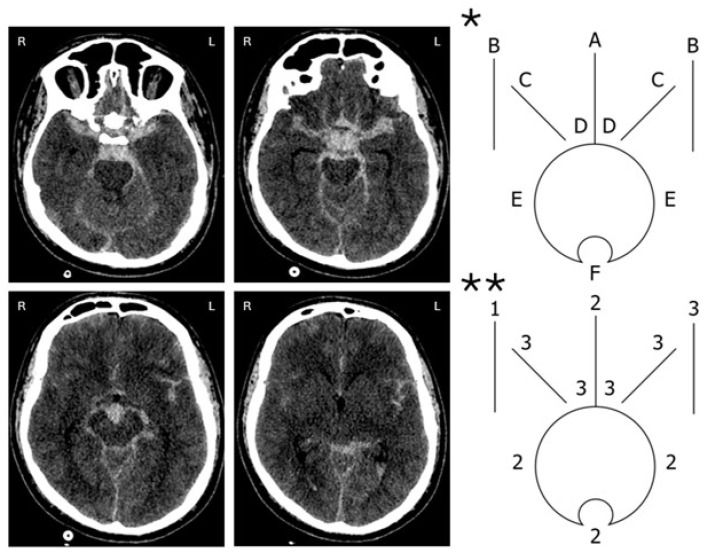
Computed tomogram of a patient with SAH. * Top diagram displays ten basal cisterns and fissures: A—frontal interhemispheric fissure; B—sylvian fissure, lateral parts; C—sylvian fissure, basal parts; D—suprasellar cistern; E—ambient cisterns; F—quadri-geminal cistern. ** Bottom diagram shows score in each cistern and fissure according to the Hijdra scale. Score on SAH totals 24.

The most complex and time-consuming is the assessment according to the Hijdra scale proposed in 1988 [67]. It involves assessing the presence of blood in each of the 10 most significant subarachnoid cisterns and four ventricles with one of four grades: 0—no blood; 1—barely visible blood; 2—partly filled with blood; and 3—completely filled with blood (Figure 2) [22,67].

Despite the difficulty of applying the scale to routine clinical practice [51,68], the literature reports its good predictive ability regarding the occurrence of cerebral vasospasm and unfavorable outcome [69].

A comprehensive analysis of the inter-rater reliability of the scales could serve as a basis for their inclusion in clinical guidelines.

### 2.3. Quality Assessment and Statistical Analysis

Risk of bias was assessed by two reviewers independently (D.D.D. and B.E.R.) using the Quality Appraisal of Reliability Studies (QAREL) checklist [70]. Two of the eleven QAREL items were considered not applicable. Item 5 was not relevant because there was no accepted reference standard in SAH severity assessment. Item 8 was not applicable as the order of raters during the review of CT images was not important. Reordering the subjects’ sequence was also unnecessary, as intra-rater reliability was out of the focus of the current research. For the remaining nine items, the criteria for choosing “yes”, “no” or “unclear” were defined by the reviewers in advance and were adjusted through joint discussion of one of the articles. Item 2 was checked “yes” if there was a description of raters’ specialization or information on the training process.

Item 4 was considered only in the context of blinding of raters that have examined subjects before their enrolment in the study (intra-rater reliability issue is not addressed). Item 11 was marked as “yes” for studies that provided point estimates and confidence intervals (CIs) of appropriate statistical metrics: Cohen’s kappa for binarized scales, weighted kappa for ordinal scales, and ICC for quantitative scales.

After evaluating all articles, the two reviewers compared their results. In case of disagreement, a consensus was reached through discussion or with the involvement of a third reviewer (G.A.V.). Since the authors of QAREL did not provide an interpretation of the summary score, the methodological quality assessment was based on previously proposed categories that consider the total number of items checked as “yes” [71]. A sum of 4 or less corresponded to low quality; 5–7 to moderate; and 8 and more to high quality.

All articles were scanned for statistical metrics of IRR: different types of kappa coefficient for qualitative scales, ICC for quantitative scales, as well as Krippendorff’s alpha for different scale types. Their point estimates and confidence intervals along with data on sample size and number of raters were summarized in the tables for each scale. Also, articles on IRR of qualitative scales were scanned for the total agreement value needed for meta-analysis. In case of its absence, we tried to find contingency tables of raters’ opinion.

The meta-analysis was conducted for Cohen’s kappa and ICC. To evaluate heterogeneity, we utilized Cochran’s Q-test, Higgins and Thompson I^2^ statistics, and heterogeneity dispersion (τ^2^) analysis. Since I^2^ values were above 50%, indicating moderate to high variability among study results, the meta-analysis was performed using a random-effects model. For the assessment of publication bias, funnel plots were built.

Given the absence of universally accepted guidelines for the interpretation of IRR coefficients, the summary estimates of Cohen’s kappa and ICC were considered in light of the most widely used grading systems: those proposed by Landis and Koch [72], Fleiss [73], and McHugh [74] for Cohen’s kappa and those proposed by Cicchetti [75] and Koo [76] for ICC.

The statistical analysis was performed using the R programming language (ver. 4.2.2) in the RStudio environment (ver. 2024.12.0, build 467), using packages “metafor” and “meta”.

## 3. Results

### 3.1. Risk of Bias Assessment

From the QAREL assessment, we found that the quality total score ranged from 3 to 8 (Table 3).

Fourteen studies were rated as having a moderate risk of bias, only one article had a total score above 8, and five articles had high risk of bias.

Information on raters’ blinding to the other raters’ findings and to clinical data (items 3 and 6, respectively) was presented in the majority of articles. Raters’ specialty and/or their training process were clearly mentioned in 63% of studies. One of the worst percentages was observed for item 11: only 7 of 20 articles applied appropriate statistical metrics and provided their point estimates and confidence intervals. Also, some reservations should be noted. All articles that analyzed IRR by means of ICC did not specify its type [20,48,59,60]. In some cases, the distribution of a scale total score was described with the median and interquartile range, and there was no mention of a non-parametric version of the coefficient [20,60]. In the case of usage of a weighted kappa only one article provided a type of weighting [51]. The most prevalent reason for checking “no” for item 11 was the absence of confidence intervals [45,46,51,64,69,77]. Also, three articles used the Kendall concordance coefficient [52,57,80]. It is a rank correlation coefficient that can give a general idea of the consistency of raters’ assessments but not their actual agreement [82]. If one of the raters systematically overestimates or underestimates the assessment, the correlation coefficient can obtain high values, while the actual IRR will remain low. This pertains to the usage of Spearman rank correlation coefficients [22]. Additionally, one of the studies [81] also evaluated the inter-rater reliability of quantitative scales (Graeb, modified Graeb, LeRoux, and IVH score) using the kappa coefficient, which is intended for categorical scales. Consequently, the results of these five studies were excluded from the subsequent meta-analysis.

Two answers were similar for all included studies. Item 7 was checked as unclear for all of them, as all authors did not mention any additional cues that were not part of the test. Also, all studies were marked as yes for item 9 as the CT images were considered as a stable variable, and their assessment did not depend on the time frame.

### 3.2. Original and Modified Fisher Scale

The systematic search revealed nine relevant articles dedicated to the original Fisher scale; three of them also contained analysis of its modified version (Table 4).

When assessing the inter-rater reliability of the original Fisher scale using Cohen’s kappa, experts’ consensus ranged from fair to good according to the Fleiss classification [73]. However, some publications presented their results without confidence intervals, which hindered proper interpretation of the findings. Studies utilizing weighted kappa indicated a wider range of values, with the lowest consistency recorded in the publication by A.H. Kramer et al. (κ = 0.45) [64] and the highest consistency found in the article by J. Claassen et al. (κ = 0.86) [45].

The assessment of inter-rater reliability of the modified Fisher scale was described in fewer publications [56,64,65]. Notably, the lower limit of the confidence interval presented in one of them was below 0.4, which corresponds to a poor level of agreement according to the Fleiss classification [73].

**Figure 3:**
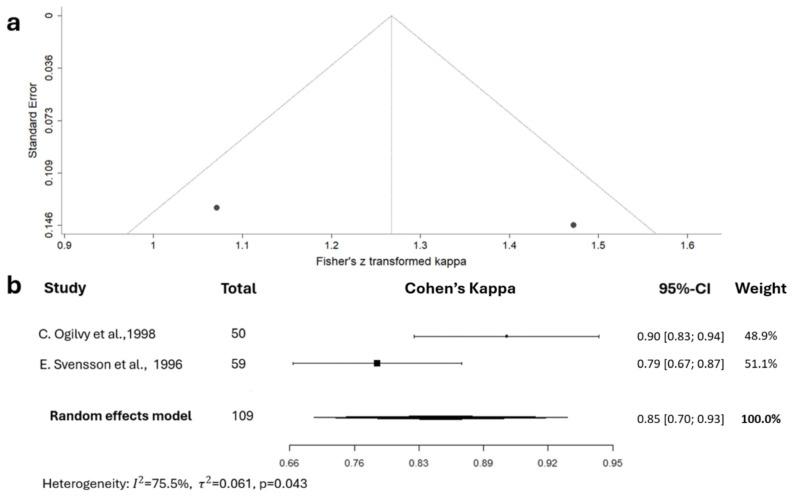
Meta-analysis of inter-rater reliability of the binarized Fisher scale: funnel plot (**a**) and forest diagram (**b**) for Cohen’s kappa. The summary estimate can be considered substantial to almost perfect according to Landis and Koch [72], fair to excellent according to Fleiss [73], and moderate to almost perfect according to McHugh [74] (E. Svensson et al., 1996 [57]; C.S. Ogilvy et al., 1998 [58]).

The total agreement value for the Fisher scale was reported only in the studies by E. Svensson et al. [57] and C.S. Ogilvy et al. [58]. However, their design was slightly different: the latter research team binarized the scale by grouping grades 0–2 and 3–4 (grade 0 was used for patients with unruptured aneurysms). To conduct a meta-analysis, we have grouped the same categories for the study of E. Svensson et al. Cohen’s kappa and total agreement value calculated based on the presented contingency table were 0.85 (95% CI 0.70, 0.93) and 93.2%, respectively. A meta-analysis on the IRR of the Fisher scale, based on these two studies, disclosed a high level of heterogeneity (I^2^ = 75.46%) (Figure 3).

The interpretation of the summary value of 0.85 (95% CI 0.70, 0.93) varies among different grading systems. According to the strictest system, proposed by McHugh [74] for health-related studies, it can be considered moderate to almost perfect.

Other studies assessing the IRR of the Fisher and modified Fisher scales did not provide total agreement values or cross-tables of raters’ opinions, which could have facilitated a manual determination of this indicator [45,46,56,64,65,78,79].

### 3.3. Original and Modified Graeb Scales

The systematic search found five publications on the IRR assessment of the original and modified Graeb scales (Table 5).

**Figure 4:**
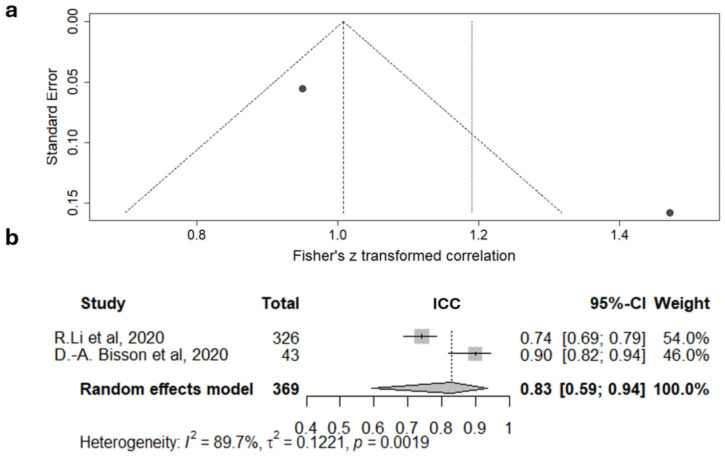
Meta-analysis of inter-rater reliability of the Graeb scale: funnel plot (**a**) and forest diagram (**b**) for the intra-class correlation coefficient (ICC). The summary estimate of the ICC can be interpreted as fair to excellent according to Cicchetti [75] and as moderate to excellent according to Koo [76] (R. Li et al., 2020 [48]; D.-A. Bisson et al., 2020 [61]).

A meta-analysis on the IRR of the original Graeb scale, based on data from two studies, revealed a high level of heterogeneity (I^2^ = 89.7%) (Figure 4).

**Figure 5:**
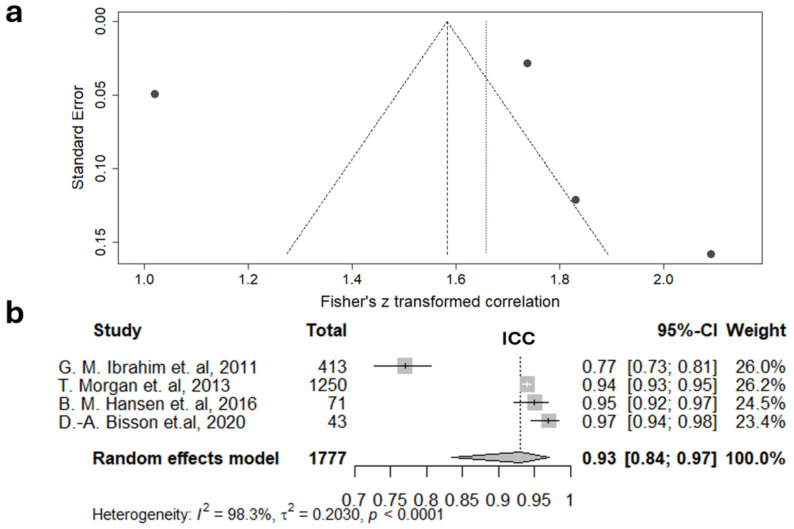
Meta-analysis of inter-rater reliability of the modified Graeb scale: funnel plot (**a**) and forest diagram (**b**) for intra-class correlation coefficient (ICC). The summary estimate of the ICC can be considered excellent according to Cicchetti [75] and good to excellent according to Koo [76]. (T. Morgan, et al., 2013 [20]; G.M. Ibrahim et al., 2011 [59]; B.M. Hansen et al., 2016 [60]; D.-A. Bisson et al., 2020 [61]).

The ICC of the modified Graeb scale was analyzed in four studies, and the meta-analysis showed even higher heterogeneity (I^2^ = 98.3%) compared to the original scale (Figure 5).

These findings indicate that IRR assessments for both the original and modified Graeb scales are inconsistent. However, the random effects model yielded ICC values of 0.83 (95% CI 0.59–0.94) and 0.93 (95% CI 0.84–0.97) for the original and modified scales, respectively. According to the classifications by Cicchetti [75] and Koo [76], the ICC for the original Graeb scale can be interpreted as fair to excellent (Cicchetti) and moderate to excellent (Koo), whereas the ICC for the modified Graeb scale is considered excellent by the Cicchetti classification and good to excellent by Koo.

### 3.4. Hijdra Scale

The systematic literature search on the IRR assessment of the Hijdra scale identified eight papers. A unique aspect of this scale is that it requires estimating blood presence in each cistern and ventricle individually. Consequently, some researchers evaluated IRR metrics separately for the cisterns and ventricles, while others focused exclusively on the total score or assessed both (Table 6).

**Figure 6:**
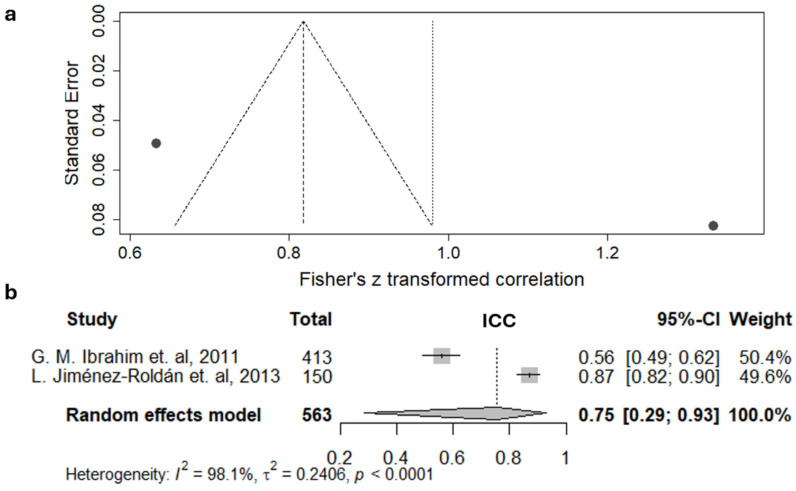
Meta-analysis of inter-rater reliability of the Hijdra scale (total score): funnel plot (**a**) and forest diagram (**b**) for the intra-class correlation coefficient (ICC). The lower bound of the confidence interval indicates a poor level of agreement according to the interpretations of both Cicchetti [75] and Koo [76]. (L. Jiménez-Roldán, 2013 [56]; G.M. Ibrahim et al., 2011 [59]).

To conduct the meta-analysis for IRR of the total score, two studies with the same design were used [56,59]. The results of these studies exhibited a very high level of heterogeneity (I^2^ = 98.1%) (Figure 6).

The ICC values varied significantly among different authors, which was the reason for the wide confidence interval when estimating the random effects model—0.75 (95% CI 0.29–0.93).

Also, Cohen’s kappa coefficients for cisterns and ventricles were analyzed separately in two articles [22,45], but as their total agreement values were not reported we were not able to conduct their meta-analysis. In addition to the IRR metrics, two studies also reported the Spearman and Pearson correlation coefficients [22,59]. Since these coefficients do not represent the degree of IRR, their values were ignored when compiling the summary table.

### 3.5. Claassen, BNI, IVH, Slice and LeRoux Scales

Also, five articles on the IRR analysis of the Claassen, BNI, IVH, Slice, and LeRoux scales were selected [46,48,56,64,65] (Table 7). However, due to a number of reasons their meta-analysis was not possible.

The Claassen scale was mentioned in two articles of a similar design that included the calculation of weighted kappa [56,64]. However, since the authors did not indicate the proportion of cases with the experts’ total agreement in the results, it was impossible to conduct a meta-analysis. Another paper estimated the IRR of this scale without weighting, using Fleiss’ kappa [65]. The IRR analysis for the BNI scale was performed twice, but it contained the assessment of different statistical metrics (weighted kappa and Fleiss’ kappa) [46,65]. The works had no information on the value of total agreement. It should be noted that, for all cases of employing weighted kappa, the type of weighting (linear or quadratic) was not indicated. The absence of original cross-tables with the distribution of expert opinions did not allow us to calculate the IRR independently. The reliability of the LeRoux and Slice scales as well as the IVH score was assessed only once [48]. The IRR provided by Sano [44] was not analyzed at all.

Also, the systematic search revealed the most recently published article devoted to the development of a new scale based on the assessment of 20 cisterns with five categories (against 10 cisterns and four ventricles with four categories in the Hijdra scale) [80]. But as the authors reported only the concordance correlation coefficient for the total score and unweighted kappa for the different cisterns, we did not include it in the table.

## 4. Discussion

The conducted systematic review and meta-analysis revealed a significant lack of robust evidence supporting the inter-rater reliability of any particular radiological scale—including the Fisher scale—or the assessment of SAH severity. Among the scales used to evaluate concomitant IVH, the mGraeb scale could be considered preferable for routine assessment.

The systematic literature review identified 20 scientific publications on the IRR assessment for the manual radiological scales used to define the severity of SAH and concomitant IVH. The quality of these articles was evaluated using the QAREL checklist. The assessment revealed that only one study had a low risk of bias. The majority of studies (70%) were characterized by a moderate level of quality and risk of bias, while five articles were rated as low quality. One of the most concerning issues was related to statistical analysis: 13 articles applied inappropriate statistical criteria or presented their results incompletely. Frequently, authors did not provide clear information regarding the blindness of raters if they have examined subjects before their enrolment in the study.

Due to various reasons, the results of only 8 of 20 articles were included in the subsequent meta-analysis, which enabled estimation of IRR of the original Fisher, Hijdra, Graeb, and mGraeb scales.

The original Fisher scale was the most frequently investigated: nine articles included its IRR assessment. Unfortunately, only two studies provided data suitable for meta-analysis, as their results included a total agreement value. In one of these studies, authors binarized the scale; thus, Cohen’s kappa of 0.85 (95% CI 0.70–0.93) can be interpreted as ranging from moderate to almost perfect agreement in the context of differentiation between grades 0–2 and 3–4. The weakness of the analysis was in its small sample size: in total, it was based on 109 CT scans only. Also, it should be noted that one of the included studies provided IRR metrics not for SAH cases only but in conjunction with CT scans of unruptured aneurysms (grade 0), and their proportion was unclear [58].

Regarding the Hijdra scale, the high level of IRR reported in several papers was not confirmed, as the random effects model was characterized by a wide confidence interval (ICC 0.75 (95% CI 0.29–0.93)). Similar to the original Fisher scale, this estimate was based on data from only two studies; therefore, additional research could significantly influence the overall assessment.

For the original and modified Graeb scales, the obtained level of agreement can be characterized as ranging from moderate to excellent for the original version (ICC 0.83 (95% CI 0.59–0.94)) and as good to excellent for the modified version (ICC 0.93 (95% CI 0.83–0.97)) [76]. Although the last result is quite promising and based on a large sample of 1777 patients, it is important to note that these scales are designed solely to assess the severity of concomitant IVH and do not evaluate SAH prevalence.

The meta-analysis of all aforementioned scales showed a lack of homogeneity of IRR estimates in the literature: I^2^ ranged from 75.5% to 98.3%. Due to the small number of studies available for each scale, the reliable assessment of publication bias via a funnel plot was not possible.

For less frequently cited scales—such as the modified Fisher scale [56,64,65], BNI [46,65], Claassen [56,64,65], LeRoux [48]—a meta-analysis was unfeasible due to differences in study design or absence of total agreement data.

Overall, insufficient reporting of statistical analysis results should be noted as a critical issue. Often, scientific publications presenting the results of the IRR assessment based on ICC did not specify the coefficient type [48,60]. Studies employing various kappa statistics often failed to provide data on the percentage of experts’ total agreement. These shortcomings prevented such studies from being included in the meta-analysis. Additionally, many studies did not specify the confidence interval for the IRR metrics, which complicated the interpretation of pairwise comparisons.

Our study has certain limitations. The systematic search was performed solely in the PubMed database; thus, some relevant studies might have been missed. However, we reviewed reference lists from all included studies and consulted with an expert in the field for recommendations. Also, there was some heterogeneity in study design of articles on IRR of scales for concomitant IVH—some studies involved clinical cases with SAH [59], while others included patients with intra-cerebral hemorrhage [20,48,60,61]. Regarding SAH etiology, most studies explicitly mentioned aneurysmal SAH as an inclusion criterion; only two articles involved samples with a small proportion of perimesencephalic hemorrhage or arteriovenous malformation [22,78], and two others did not specify the cause of SAH [57,80].

It is worth noting that the heterogeneity among the aims of the included studies—for example, regarding the evaluation of the Fisher scale’s informativeness in risk assessment of poor clinical outcome [65] or vasospasm [64]—should not be considered a limitation. The gradations of a scale should be interpreted consistently, regardless of the further purpose of the study.

In terms of potential clinical recommendations derived from this analysis, it can be concluded that there is currently insufficient robust evidence to confidently incorporate any of the existing scales for SAH severity assessment into clinical practice guidelines. Even for the Fisher scale, the most widely used and oldest scale, pooled analysis was available only in the context of distinguishing grades 0–2 and 3–4. Regarding scales used to evaluate concomitant IVH, the mGraeb scale appears to be the most reliable. Although its confidence interval overlaps with that of the original scale, its IRR can be interpreted as good to excellent and is supported by validation across a sizable sample.

To sum up, there is a strong demand for additional studies to assess the IRR of the aforementioned scales. Incorporating scales with insufficient evidence of their reliability into clinical guidelines could lead to adverse outcomes. This issue is particularly relevant given the rapid development of machine learning methods and automated blood segmentation algorithms [83,84,85]. The performance of such algorithms often relies on these scales as reference standards; therefore, their reliability and reproducibility are crucial.

## 5. Conclusions

Despite the significant socioeconomic burden of SAH, there is currently no unified approach to assessing its severity in clinical guidelines. This gap may be partly due to the lack of high-quality studies analyzing the inter-rater reliability of various scales. The conducted meta-analysis has revealed considerable variability in the IRR assessments for the original Fisher and original and modified Graeb, as well as the Hijdra scale, across different studies. The original Fisher scale, which is most frequently referenced in the literature, demonstrated moderate to almost perfect reliability (Cohen’s kappa 0.85 (95% CI 0.70, 0.93)). However, this assessment was possible only after binarization of the scale and based on a relatively small sample size. The Hijdra scale was characterized by a wide confidence interval, with a lower limit of 0.29 indicating a poor level of IRR. The highest IRR metrics were observed for the modified Graeb scale, which assesses concomitant IVH, though it did not differ statistically from the original one (ICC 0.93 (95% CI 0.84–0.97) and 0.83 (95% CI 0.59–0.94), respectively). While these assessments were obtained on a limited number of studies with similar design, meta-analysis of the modified Fisher, BNI, Claassen, and LeRoux scales was not feasible for all. To establish a more comprehensive evidence base, further research evaluating the IRR of manual scales for SAH assessment is essential.

## Figures and Tables

**Table 1:** Fisher, modified Fisher, Claassen and BNI scales.

Grade	Fisher Scale	mFisher Scale	Claassen Scale	BNI Scale
0	-	No blood detected	No blood detected	-
1	No blood detected	Focal or diffuse thin SAH, no IVH	Minimal/thin SAH, no IVH in both lat ventricles	No visible SAH
2	Diffuse deposition or thin layer. All vertical layers of blood < 1 mm thick	Focal or diffuse thin SAH, with IVH	Minimal/thin SAH, with IVH in both lat ventricles	Maximum SAH thickness ≤ 5 mm
3	Localized clots and/or vertical layers of blood > 1 mm thick	Thick SAH present, no IVH	Thick SAH, no IVH in both lat ventricles	Maximum SAH thickness >5 to ≤10 mm
4	Intra-ventricular or intra-parenchymal blood present	Thick SAH present, with IVH	Thick SAH, with IVH in both lat ventricles	Maximum SAH thickness >10 to ≤15 mm
5	-	-	-	Maximum SAH thickness >15 mm

BNI—Barrow Neurological Institute, SAH—subarachnoid hemorrhage, IVH—intra-ventricular hemorrhage.

**Table 2:** Scales for concomitant IVH assessment: Graeb scale, IVH score, LeRoux and modified Graeb scale.

Score		Graeb Scale	IVH Score	LeRoux Scale	mGraeb Scale
Lateral ventricles	0	no blood present	no blood or small amount of blood	no blood present	no blood present
	1	trace of blood or mild bleeding	up to a third filled with blood	trace of blood	≤25% filled
	2	less than half of the ventricle filled with blood	one to two thirds filled with blood	less than half a single ventricle filled with blood	>25 to ≤50% filled
	3	more than half of ventricle filled with blood	mostly or completely filled with blood	more than half a single ventricle filled with blood	>50 to ≤75% filled
	4	ventricle filled with blood and expanded	-	entire ventricle filled and expanded with blood	>75 to 100% filled
Third and fourth ventricles	0	no blood present	no blood present	no blood present	no blood present
	1	blood present, ventricle size normal	partially or completely filled with blood	trace of blood	-
	2	ventricle filled with blood and expanded	-	less than half a single ventricle filled with blood	≤50% filled
	3	-	-	more than half a single ventricle filled with blood	-
	4	-	-	entire ventricle filled and expanded with blood	>50% filled
Hydrocephalus	0	-	absent	-	-
	1	-	present	-	-
Temporal/posterior tips	0	-	-	-	no blood present
	1	-	-	-	≤50% filled
	2	-	-	-	>50% filled
Expanded ventricle or tip	0	-	-	-	no expansion
	1	-	-	-	expanded ventricle or horn
Maximum score		12	23	16	32

IVH—intra-ventricular hemorrhage.

**Table 3:** Quality appraisal of included studies using QAREL checklist.

Study	QAREL Items											Risk of Bias (Quality)
	1	2	3	4	5	6	7	8	9	10	11	
A Hijdra et al., 1990 [22]	Y	?	?	?	NA	?	?	NA	Y	Y	N	3/11 High (Low)
E. Svensson et al., 1996 [57]	?	Y	Y	Y	NA	Y	?	NA	Y	Y	N	6/11 Moderate (Moderate)
C.S. Ogilvy et al., 1998 [58]	N	?	?	?	NA	?	?	NA	Y	Y	N	2/11 High (Low)
M. Jagt et al., 2000 [77]	Y	?	Y	?	NA	Y	?	NA	Y	Y	N	5/11 Moderate (Moderate)
J. Claassen et al., 2001 [45]	N	?	Y	?	NA	Y	?	NA	Y	Y	N	4/11 High (Low)
A. Norden et al., 2006 [78]	?	Y	?	?	NA	?	?	NA	Y	Y	N	3/11 High (Low)
A.H. Kramer et al., 2008 [64]	N	Y	Y	N	NA	Y	?	NA	Y	Y	N	5/11 Moderate (Moderate)
G.M. Ibrahim et al., 2011 [59]	Y	Y	?	NA	NA	?	?	NA	Y	Y	Y	5/11 Moderate (Moderate)
D.A. Wilson et al., 2012 [46]	N	Y	Y	?	NA	Y	?	NA	Y	Y	N	5/11 Moderate (Moderate)
T.C. Morgan et al., 2013 [20]	Y	?	Y	Y	NA	Y	?	NA	Y	Y	Y	7/11 Moderate (Moderate)
L. Jiménez-Roldán, 2013 [56]	Y	?	Y	?	NA	Y	?	NA	Y	Y	Y	6/11 Moderate (Moderate)
U.T. Siddiqui et al., 2014 [79]	N	Y	Y	?	NA	Y	?	NA	Y	Y	Y	6/11 Moderate (Moderate)
B.M. Hansen et al., 2016 [60]	Y	Y	Y	Y	NA	Y	?	NA	Y	Y	Y	8/11 Low (High)
P.Y.M. Woo et al., 2017 [65]	Y	Y	Y	?	NA	Y	?	NA	Y	Y	N	6/11 Moderate (Moderate)
R. Li et al., 2020 [48]	?	Y	Y	Y	NA	Y	?	NA	Y	Y	Y	7/11 Moderate (Moderate)
M.J. Kole et al., 2020 [51]	Y	?	Y	?	NA	Y	?	NA	Y	Y	N	5/11 Moderate (Moderate)
D.-A. Bisson et al., 2020 [61]	Y	Y	Y	?	NA	Y	?	NA	Y	Y	Y	7/11 Moderate (Moderate)
C. Melinosky, 2021 [52]	N	Y	Y	Y	NA	Y	?	NA	Y	N	N	5/11 Moderate (Moderate)
E. Slonimsky et al., 2022 [80]	Y	Y	Y	Y	NA	Y	?	NA	Y	Y	N	7/11 Moderate (Moderate)
I. Haffaf et al., 2025 [81]	Y	?	Y	?	NA	Y	?	NA	Y	?	N	4/11 High (Low)

QAREL—Quality Appraisal for Reliability Studies, Y—yes, NA—not applicable, N—no, ?—unclear.

**Table 4:** Summary table for articles on the IRR of the original and modified Fisher scales.

Scale	Study	Statistical Method	IRR (95% CI)	Total Agreement, %	Sample Size	Number of Raters
Fisher	E. Svensson et al., 1996 [57]	Cohen’s kappa	0.63	76	59	4 *
	C.S. Ogilvy et al., 1998 [58]	Cohen’s kappa	0.9	96	50	2
	J. Claassen et al., 2001 [45]	Weighted kappa	0.86	-	32	2
	A. Norden et al., 2006 [78]	Cohen’s kappa	0.55 (0.43–0.67)	-	131	3 *
	A.H. Kramer et al., 2008 [64]	Weighted kappa	0.45	-	40	3 *
	D.A. Wilson et al., 2012 [46]	Weighted kappa	0.51	-	30	3 *
	L. Jiménez-Roldán et al., 2013 [56]	Weighted kappa	0.64 (0.50–0.76)	-	150	2
	U.T. Siddiqui et al., 2014 [79]	Weighted kappa	0.702 (0.637–0.743)	-	35	4 *
	P.Y.M. Woo et al., 2016 [65]	Fleiss’ kappa	0.53 (0.48–0.59)	-	165	4
Modified Fisher	A.H. Kramer et al., 2008 [64]	Weighted kappa	0.59	-	40	3 *
	L. Jiménez-Roldán et al., 2013 [56]	Weighted kappa	0.59 (0.47–0.70)	-	150	2
	P.Y.M. Woo et al., 2016 [65]	Fleiss’ kappa	0.42 (0.37–0.46)	-	165	4

IRR—inter-rater reliability, *—analysis was based on pair comparison of raters’ assessments.

**Table 5:** Summary table for articles on IRR of the original and modified Graeb scales.

Scale	Study	ICC (95% CI)	Sample Size	Number of Raters
Graeb	R. Li et al., 2020 [48]	0.74 (0.41–0.89)	326	2
	D.-A. Bisson et al., 2020 [61]	0.90 (0.80–0.95)	43	2
mGraeb	G.M. Ibrahim et al., 2011 [59]	0.77 (0.72–0.81)	413	2
	T. Morgan et al., 2013 [20]	0.94 (0.93–0.95)	1250	2
	B.M. Hansen et al., 2016 [60]	0.95 (0.92–0.97)	71	2
	D.-A. Bisson et al., 2020 [61]	0.97 (0.84–0.99)	43	2

ICC—intra-class correlation coefficient.

**Table 6:** Summary table for articles on IRR of the Hijdra scale.

Study	Statistical Method	IRR (95% CI)			Sample Size	Number of Raters
		Total Score	Ventricles	Cisterns		
Hijdra et al., 1990 [22]	Fleiss’ kappa	-	0.74	0.65	182	3 *
	Weighted kappa	-	0.81	0.78		
M. Jagt et al., 2000 [77]	Cohen’s kappa	-	-	0.66	159	2
J. Claassen et al., 2001 [45]	Weighted kappa	-	0.83	0.61	32	2
A. Norden et al., 2006 [78]	Fleiss’ kappa	0.75 (0.65–0.85)	0.68 (0.55–0.81)	0.81 (0.73–0.90)	131	3 *
G.M. Ibrahim et al., 2011 [59]	ICC	0.56 (0.49–0.62)	-	-	413	2
L. Jiménez-Roldán et al., 2013 [56]	Weighted kappa	0.82 (0.77–0.87)	-	-	150	2
	ICC	0.87 (0.71–0.93)	0.92 (0.89–0.94)	0.84 (0.64–0.91)		
P.Y.M. Woo et al., 2016 [65]	Fleiss’ kappa	0.66 (0.62–0.71)	-	-	165	4
M.J. Kole et al., 2020 [51]	Weighted kappa (quadratic)	0.928	-	-	431	2

IRR—inter-rater reliability, CI—confidence interval, ICC—intra-class correlation coefficient, *—analysis is based on pair comparison of rater assessments.

**Table 7:** Summary table for articles on the IRR of the Claassen, BNI, IVHS, LeRoux and Slice scales.

Scale	Study	Statistical Method	IRR (95% CI)	Sample Size	Number of Raters
Claassen	A.H. Kramer et al., 2008 [64]	Weighted kappa	0.64	40	3 *
	L. Jiménez-Roldán et al., 2013 [56]	Weighted kappa	0.61 (0.49–0.73)	150	2
	P.Y.M. Woo et al., 2016 [65]	Fleiss’ kappa	0.38 (0.33–0.42)	165	4
BNI	D. Wilson et al., 2012 [46]	Weighted kappa	0.65	30	3 *
	P.Y.M. Woo et al., 2016 [65]	Fleiss’ kappa	0.2 (0.17–0.24)	165	4
IVH score	R. Li et al., 2020 [48]	ICC	0.76 (0.50–0.90)	413	2
LeRoux	R. Li et al., 2020 [48]	ICC	0.85 (0.61–0.94)	413	2
Slice	R. Li et al., 2020 [48]	ICC	0.95 (0.88–0.98)	413	2

IRR—inter-rater reliability, CI—confidence interval, ICC—intra-class correlation coefficient, *—pair comparison of rater assessments.

## Data Availability

The authors confirm that the data supporting the findings of this study are available within the article and its Supplementary Materials.

## Supplementary Materials

The following supporting information can be downloaded at: https://www.mdpi.com/article/10.3390/jcm15082899/s1, File S1: Review protocol; Table S1: PRISMA 2020 checklist; Table S2: PRISMA 2020 for Abstracts Checklist.

## Abbreviations

The following abbreviations are used in this manuscript:

BNI	Barrow Neurological Institute
CI	Confidence interval
CT	Computed tomography
ICC	Intra-class correlation coefficient
IRR	Inter-rater reliability
IVH	Intra-ventricular hemorrhage
PRISMA	Preferred Reporting Items for Systematic Reviews and Meta-Analyses
QAREL	Quality Appraisal of Reliability Studies
SAH	Subarachnoid hemorrhage
