# Supplementary material for: Inter-Rater Reliability of Subarachnoid Hemorrhage Radiological Grading Scales: A Systematic Review and Meta-Analysis
Source: J Clin Med. 2026 Apr 10;15(8):2899. doi: 10.3390/jcm15082899 (PMC13116611; doi:10.3390/jcm15082899)
Supplement: Supplementary file 1 [file jcm-15-02899-s001.zip › Supplementary File S1_Review Protocol.pdf]

Supplementary File S1.

Review protocol:
"Inter-rater reliability of subarachnoid hemorrhage radiological grading scales: a systematic review and meta-analysis"

Daria Dmitrievna Dolotova[1,2], Tatyana Alexandrovna Solominova[1], Natalia Alexeevna Polunina[3], Evgenia Romanovna Blagosklonova[2], Natalya Sergeevna Plyusova[4], Ganipa Ramazanovich Ramazanov[4], Rustam Shakhismailovich Muslimov[4], Maxim Vladimirovich Solominov[2], and Andrey Vasilevich Gavrilov[2,5]

1 Pirogov Russian National Research Medical University, Research and Clinical Institute for Pediatrics Named after Yuri Veltischev, 125412 Moscow, Russia; dolotova_dd@rsmu.ru
2 Gammamed-Soft, Ltd., Research Department, 127473 Moscow, Russia; tatiana.solominova@gammamed.ru, evgenia.blagosklonova@gammamed.ru, maksim.solominov@gammamed.ru
3 Pirogov Russian National Research Medical University, Department of Fundamental Neurosurgery, 117513 Moscow, Russia; polunina_na@rsmu.ru
4 Sklifosovsky Research Institute for Emergency Medicine, Moscow Health Department, 129090 Moscow, Russia; plyusovans@sklif.mos.ru, ramazanovgr@sklif.mos.ru, muslimovrsh@sklif.mos.ru
5 Lomonosov Moscow State University, Scobeltsyn Nuclear Physics Research Institute, 119991 Moscow, Russia; andrey.gavrilov@gammamed.ru

Background

Describe the population and condition or phenomenon of interest and contextualize it. In other words, describe what this review is about.

Subarachnoid hemorrhage (SAH) is a critical cerebrovascular event characterized by bleeding into the subarachnoid space, often resulting from ruptured aneurysms. It affects a relatively young population and carries high mortality rates. Accurate assessment of SAH severity is crucial for prognosis and management, with computed tomography (CT) being the primary diagnostic tool. Various radiological grading scales have been developed to evaluate the extent of SAH and associated conditions like intraventricular hemorrhage (IVH). Inter-rater reliability (IRR) of these scales is fundamental for ensuring consistent clinical decision-making, yet a comprehensive synthesis of existing evidence on IRR across different scales is lacking. This review aims to address this gap by systematically evaluating and comparing the IRR of these grading scales.

Objective

Describe the justification for this review. In other words, describe why this review/the information it collects is important.

The primary aim of this review is to systematically identify, evaluate, and synthesize existing studies that report on the IRR of radiological grading scales used for assessing SAH and IVH on computed tomography. Understanding the consistency of these scales is essential for their clinical adoption and optimal patient management. This review will inform clinicians about the most reliable tools available, thereby improving diagnosis and treatment planning.

Review question

**Full review question**<br>Provide the full review question in sentence format, and then break up the question according to the SDMO framework.	
What is the inter-rater reliability of radiological grading scales used in the assessment of subarachnoid hemorrhage (SAH) and intraventricular hemorrhage (IVH) on computed tomography (CT) images, in terms of inter-rater agreement?	
**Types of Studies**	All studies regardless of design that include quantitative assessment of IRR of radiological grading scales for SAH and/or IVH.
**Types of Data**	Studies that analyse non-contrast brain computed tomography (CT) images of patients with SAH and IVH.
**Types of Methods**	Any scales used to describe the severity of SAH or IVH (e.g., Fisher, Hijdra, Graeb, modified Fisher, etc.), regardless of their subsequent use (for predicting complications, outcomes, or other clinical parameters).
**Types of Outcome Measures**	Statistical measures of IRR, such as kappa coefficients, intra-class correlation coefficients (ICCs), or other metrics.

Search strategy

**Databases**<br>List the bibliographic databases to be searched.<br>If you are unsure look at the list of databases on the E-resources tab of your subject guide www.reading.ac.uk/library/subjects and try scoping searches with your key terms to decide which ones are relevant.
• PubMed
**Search terms**<br>What are your key search terms? Pick out the key words and think about alternatives you will need to include in your search.<br>For guidance on constructing a search see: libguides.reading.ac.uk/database-searching

The following search terms will be used ((("subarachn*" OR "intraventricul*" OR "intracerebr*") AND ("hemorrhage" OR "haemorrhage"OR"extension")) OR "aneurysmal" OR "aSAH" OR "SAH" OR "IVH" OR "ICH" OR "DCI" OR (("subarachoid" OR "extravasated" OR "ventricular" OR "intraventricular" OR "cisternal" OR "intracerebral" OR "subarachnoid") AND ("blood" OR ("hemorrhage" OR "haemorrhage")))) AND ((("radiological" OR "CT" OR "computed tomogram" OR "computed tomography") OR ("Fisher" OR "original Fisher" OR "mFisher" OR "modified Fisher" OR "Graeb" OR "original Graeb" OR "Modified Graeb" OR "mGraeb" OR "mGS" OR "Hijdra" OR "modified Hijdra" OR "Claassen" OR "BNI" OR "LeRoux" OR "Slice") OR ("grading" OR "quantitative" OR "new" OR "comprehensive")) AND ("scale" OR "score" OR "featur*" OR "grad*" OR "method" OR "scoring system" OR "sum score")) AND (("inter-observer" OR "interobserver" OR "inter-rater" OR "interrater" OR "inter-expert" OR "inter-") AND ("agreement" OR "reliability" OR "variability") OR ("ICC" and "kappa"))

Identifying other useful sources

Outline any other ways you will find useful sources, such as hand searching contents lists of key journals, looking at relevant websites, looking at references and citations for relevant articles.

Scanning references in retrieved articles

Reviewing citations

Eligibility criteria

Give more detail about your SDMO concepts by explicitly stating what would and would not meet inclusion.

SDMO	Inclusion Criteria	Exclusion Criteria
Types of Studies	• All scientific publications, regardless of study design, that include a quantitative assessment of inter-rater reliability (IRR) of radiological grading scales for SAH and/or IVH.	• Conference abstracts, commentaries, editorials, case reports, case series, simulation studies using non-human or synthetic data, and studies lacking quantitative IRR data, articles without full-text,
Types of Data	Non-contrast brain CT scans from:<br>• Human patients with SAH and/or IVH confirmed by CT;<br>• All gender;<br>• Over 18 years old;<br>• All races and ethnicity.	• Non-human CT scans<br>• Studies without evidence of SAH or IVH on CT images<br>• Studies using imaging modalities other than CT (e.g., MRI).
Types of Methods	• CT grading scales used for assessing the severity of SAH or IVH (e.g., original and modified Fisher scale, original and modified Graeb scale, etc.)	• Scales based on non-radiological data;<br>• Radiological scales based on any modality other from CT;<br>• Radiological scales proposed for other types of hemorrhage or unrelated pathology

Types of Outcome Measures	• Studies reporting IRR metrics including, but not limited to, Cohen's kappa, weighted kappa, Fleiss's kappa, intra-class correlation coefficients (ICC)	• Studies reporting results of any other types of analyses without investigation of inter-rater reliability (e.g., descriptive statistics, other types of reliability)

Additional limits

Outline any other limits you impose e.g. language, publication type, study design (e.g. randomised controlled trials)?

We will include only articles written in English.

Study quality assessment

What criteria will you use to assess methodological quality? How will quality assessment be performed?

The quality of each included study will be evaluated independently by two authors using the QAREL checklist. Discrepancies will be resolved through discussion, and, if necessary, by involving a third author.

Data extraction & synthesis

Outline the procedures you intend to use to analyse and summarise the study results, including whether or not you intend to carry out meta-analyses. How will you extract the data and how will it be analysed and summarised (statistical or narrative)?

Data extraction (selection and coding)

Two independent authors will screen the database search results, assessing articles based on the title and abstract according to the pre-defined inclusion and exclusion criteria. Articles whose eligibility is unclear at this stage will proceed to full-text review. The full texts of the remaining papers will be retrieved and independently assessed by both authors to extract key characteristics of studies, including:

- Sample details (sample size, types of hemorrhage).
- Imaging parameters (CT scanner details, timing of scans).
- Raters' characteristics (number of raters, expertise, training).
- Description of the grading scales used.
- IRR metrics (kappa, ICC, etc.), including their confidence intervals.
- Key findings related to IRR.

Any disagreements will be resolved through discussion, with a third reviewer available to arbitrate.

Strategy for data synthesis

The reporting of the systematic review will be guided by the standards of the Preferred Reporting Items for Systematic Review and Meta-Analysis (PRISMA) Statement and will be displayed using flow diagram. The quality assessment will be performed with QAREL checklist by two authors independently. Disagreement will be solved with through joint discussion with the participation of a third author.

- If sufficient homogeneous data are available (e.g., multiple studies reporting kappa coefficients for a specific scale), a meta-analysis will be conducted to pool IRR estimates.
- Heterogeneity will be assessed with I^2 statistics.
- Analyses will be conducted based on scale type.
- Publication bias will be explored via funnel plots if enough studies are available.

Adapted from a Review Protocol Template by Sarah Visintini which is licensed under a Creative Commons Attribution-NonCommercial-ShareAlike 4.0 International License.
